# Description of *Mycobacterium pinniadriaticum* sp. nov., isolated from a noble pen shell (*Pinna nobilis*) population in Croatia

**DOI:** 10.3389/fmicb.2023.1289182

**Published:** 2023-12-15

**Authors:** Silvio Špičić, Sanja Duvnjak, Bojan Papić, Irena Reil, Snježana Zrnčić, Željko Mihaljević, Šimun Naletilić, Ivana Giovanna Zupičić, Gordan Kompes, Boris Habrun, Ivana Mareković, Maja Zdelar-Tuk

**Affiliations:** ^1^Laboratory for Bacterial Zoonosis and Molecular Diagnostics of Bacterial Diseases, Department of Bacteriology and Parasitology, Croatian Veterinary Institute, Zagreb, Croatia; ^2^Veterinary Faculty, Institute of Microbiology and Parasitology, University of Ljubljana, Ljubljana, Slovenia; ^3^Laboratory for Fish Pathology, Department for Pathological Morphology, Croatian Veterinary Institute, Zagreb, Croatia; ^4^Laboratory for Pathology, Department for Pathological Morphology, Croatian Veterinary Institute, Zagreb, Croatia; ^5^Laboratory for General Bacteriology and Mycology, Department of Bacteriology and Parasitology, Croatian Veterinary Institute, Zagreb, Croatia; ^6^Department of Clinical and Molecular Microbiology, University Hospital Centre Zagreb, Zagreb, Croatia

**Keywords:** noble pen shell, mass mortality event, Croatia, *Mycobacterium*, Adriatic Sea, average nucleotide identity

## Abstract

**Introduction:**

Shortly before the mass mortality event of the noble pen shell (*Pinna nobilis*) population in the south-eastern Adriatic coast, two rapidly growing *Mycobacterium* strains CVI_P3^T^ (DSM 114013 T, ATCC TSD-295 T) and CVI_P4 were obtained from the organs of individual mollusks during the regular health status monitoring.

**Methods:**

The strains were identified as members of the genus *Mycobacterium* using basic phenotypic characteristics, genus-specific PCR assays targeting the *hsp65* and 16S rRNA genes and the commercial hybridization kit GenoType Mycobacterium CM (Hain Lifescience, Germany). MALDI-TOF mass spectrometry did not provide reliable identification using the Bruker Biotyper Database.

**Results and discussion:**

Genome-wide phylogeny and average nucleotide identity (ANI) values confirmed that the studied strains are clearly differentiated from their closest phylogenetic relative *Mycobacterium aromaticivorans* and other validly published *Mycobacterium* species (ANI ≤ 85.0%). The type strain CVI_P3^T^ was further characterized by a polyphasic approach using both phenotypic and genotypic methods. Based on the phenotypic, chemotaxonomic and phylogenetic results, we conclude that strains CVI_P3^T^ and CVI_P4 represent a novel species, for which the name *Mycobacterium pinniadriaticum* sp. nov. is proposed.

## 1 Introduction

The genus *Mycobacterium* (*M.*) was first named in 1896 by Lehmann and Neumann based on phenotypic characteristics such as the presence of mycolic acid in the cell wall, aerobic growth and bacillary cell shape. Currently, the genus comprises nearly 200 validly published species (Parte et al., 2020), including major human pathogens such as *M. tuberculosis* and *M. leprae*. Based on phenotypic and phylogenomic data, the genus can be divided into rapid and slow growers. A recent study on the genome-wide phylogeny of the genus *Mycobacterium* proposed to split the genus into four new genera (*Mycolicibacterium*, *Mycolicibacter*, *Mycolicibacillus*, and *Mycobacterioides*) and an emended genus *Mycobacterium* (Gupta et al., 2018). However, another recent study using multiple genome-based methods showed that the new genera overlap and therefore suggested that they should be combined into a single genus named *Mycobacterium* (Meehan et al., 2021). Based on clinical manifestation, most *Mycobacterium* species are non-tuberculous mycobacteria (NTM), many of which are important opportunistic pathogens and can be found in various niches and environments (Fedrizzi et al., 2017). To date, many new *Mycobacterium* species have been described, which were first described in different environments such as natural water ecosystems (Zhang et al., 2013; Fogelson et al., 2018; Nouioui et al., 2018) and artificial water systems (Shahraki et al., 2017).

The noble pen shell *Pinna (P.) nobilis* (Linnaeus, 1758) is endemic and the largest bivalve of the Mediterranean Sea, inhabiting soft-bottom coastal areas and seagrass meadows. Occasionally, it also thrives on unvegetated bottoms, maerl beds and boulders (Zavodnik et al., 1991; García-March et al., 2002; Kersting and García-March, 2017; Öndes et al., 2020). Currently, the pen shell populations in the Mediterranean Sea are seriously endangered by *Haplosporidium (H.) pinnae*, a haplosporidian endoparasite that causes mass mortality events (MMEs). Mortality was first reported in the Western Mediterranean, off the coasts of Spain and France in late 2016 (Darriba, 2017; Vázquez-Luis et al., 2017), later expanding along the shores of Turkey, Greece, Albania, and Croatia (Cabanellas-Reboredo et al., 2019; Carella et al., 2019; Panarese et al., 2019; Tiscar et al., 2019; Čižmek et al., 2020; Šarić et al., 2020; Mihaljević et al., 2021). In just 5 years, these MMEs were observed in all parts of the Mediterranean Sea. Although *H. pinnae* is believed to be the main cause of mortality, mycobacteria have also been detected in the affected shell tissue using genetic methods (Carella et al., 2019, 2020; Čižmek et al., 2020; Lattos et al., 2020; Šarić et al., 2020; Mihaljević et al., 2021). To our knowledge, *Mycobacterium* sp. has never been successfully cultured from the tissue of healthy or diseased *P. nobilis*. Finally, the hypothesis that MMEs are caused by multiple pathogens remains uncertain (Scarpa et al., 2020). A pronounced effect of anthropogenic factors related to climate change suggests multifactorial disease as a possible explanation for the MMEs of *P. nobilis* (Šarić et al., 2020; Scarpa et al., 2020). Here, we describe a novel mycobacterial species for which we propose the name *Mycobacterium pinniadriaticum*. Because strains CVI_P3^T^ and CVI_P4 were both obtained from a single tissue (gills and mantle, respectively) collected a few weeks before the MMEs in Mljet National Park (Croatia) in 2019, its possible involvement in mass mortality events of pen shells remains to be elucidated.

## 2 Materials and methods

One of the most important habitats of *P. nobilis* in the southern Eastern Adriatic Sea is the Mljet National Park, which is a very productive and biodiverse marine ecosystem and is part of the Natura 2000 European Network of Protected Areas (code HR5000037). It has two lake-like inlets: the Small Lake and the Big Lake. In April 2019, health status control was conducted at five sites in Mljet National Park located in the southeast Adriatic Sea. Healthy shells were collected in April 2019 at a sea temperature of 17.2°C. The deepest points along the transects were from 3.8 m to 7.3 m (Mihaljević et al., 2021). Five sampling sites were selected according to the most abundant pen shell populations. Sampling sites 1 and 2 were located in the Small Lake (Supplementary Figure S1), a lake-like inlet connected by a shallow, narrow channel to the Big Lake (sampling site 3), which was connected to the open sea by a slightly deeper, wider channel. Two additional sampling sites besides these lake-like inlets were Gonoturska Bay (sampling site 4) and Cape Lenga (sampling site 5) (Mihaljević et al., 2021). Five healthy individuals were collected, one for each sampling site (Mihaljević et al., 2021). Samples of digestive glands, mantles and gills were collected for bacteriological examination. All samplings were conducted in April 2019 with permission of the Croatian Ministry of Environmental Protection and Energy (CLASS UP/1-612-17/18-48/172; No. 517-05-1-1-18-4 of 21 December 2018 and CLASS UP/1-612-07/19-48/193; No. 517-05-1-1-19-3 of 11 September 2019).

**Figure 1:**
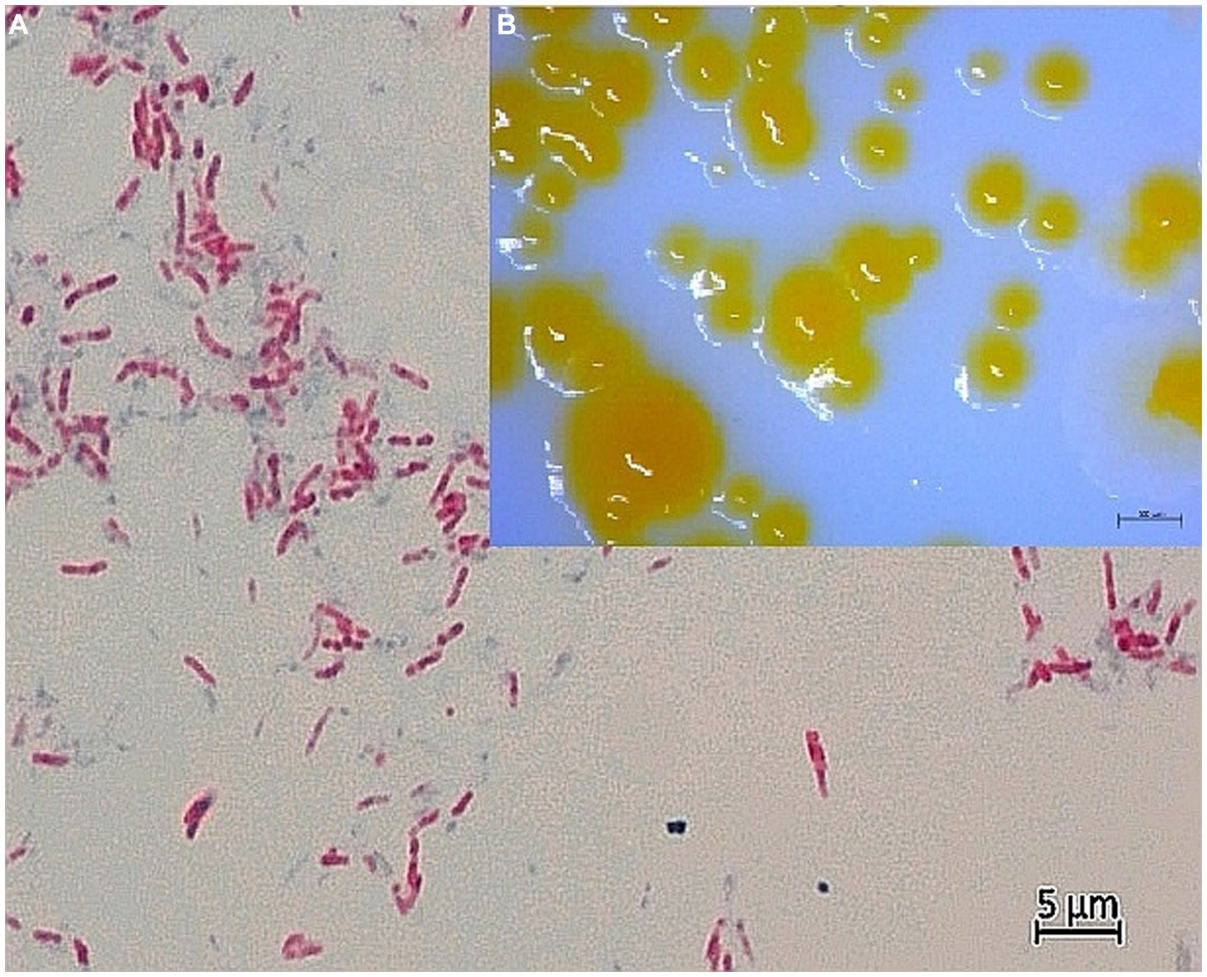
**(A)** Light microscopy of Ziehl-Neelsen-stained strain CVI_P3^T^ at 1000x magnification with oil immersion. Acid-fast rods, separate and in clusters, are visible. Smear was prepared from bacterial colonies in pure culture on Löwenstein–Jensen agar supplemented with pyruvate. The photo was taken by AxioCam MRC5 with Axio Imager.A2 microscope (CarlZeiss, AG, Germany). **(B)** Colony morphology of CVI_P3^T^ bacterial colonies in a pure culture grown on Löwenstein–Jensen agar supplemented with pyruvate. The image was taken after 14 days of growth at room temperature (24 ± 1°C) using the stereomicroscope Discovery v20 (Carl Zeiss AG, Germany).

Samples were homogenized, concentrated and decontaminated as described previously (Kent and Kubica, 1985). The material was inoculated on three standard nutrient media: Löwenstein-Jensen (LJ) slant supplemented with pyruvate, LJ slant supplemented with glycerol, and Stonebrink slant. The inoculated media were incubated at 28, 37, and 45°C under light and dark conditions. Each sample was decontaminated using 5% oxalic acid for 10 min at room temperature. For each sample, 200 μL of homogenized suspension was inoculated on standard nutrient media as described previously. Slants were checked for growth twice a week for 8 weeks. All grown colonies underwent Ziehl-Neelsen (ZN) and Gram staining. Acid-fast bacilli indicative of mycobacteria were subcultured (Figure 1). Growth rate, ability to grow at different temperatures and colony morphology were recorded. Growth, biochemical and phenotypic characterization were performed in parallel at the Croatian Veterinary Institute in Zagreb, Croatia and the Leibniz-Institut Deutsche Sammlung von Microorganismen und Zellkulturen GmbH (DSMZ), Braunschweig, Germany.

Mycolic acid analysis was performed at DSMZ on cultures grown at 28°C for 9 days on M.65 media plates. Mycolic acids were extracted according to the protocol described previously (Vilchèze and Jacobs, 2007) by hydrolyzing mycolic acids from the cell wall by saponification. If the mero-chain contains wax-esters, these are hydrolyzed, resulting in the formation of dicarboxy mycolic acids. Mycolic acids were identified using mass spectrometry-based on the exact masses of mycolic acids. The relative abundance of the mycolic acids was calculated based on the sum of the identified mycolic acids. To test for the presence of long-chain alcohols, the fatty acid methyl ester mixtures were separated by gas chromatography and detected by a flame ionization detector using Sherlock Microbial Identification System (MIS) version 6.4 and myco6 database (MIDI, Microbial ID, Newark, DE, United States). Peaks were automatically integrated and fatty acid names and percentages were calculated by the MIS Standard Software (Microbial ID). Obtained fatty acid profiles were compared with the profiles of other rapidly growing mycobacteria (Vuorio et al., 1999; Hennessee et al., 2009; Tortoli et al., 2009; Musser et al., 2022).

Antimicrobial susceptibility testing (AST) was performed by broth microdilution method using two commercial AST plates, namely Sensititre Myco SLOMYCO (Thermo Scientific) and RAPMYCO (TREK Diagnostic Systems, East Grinstead, UK). Plates were incubated at 36 ± 1°C and 25°C for 7 days (up to 14 days if bacterial growth was poor).

MALDI-TOF mass spectrometry analysis of the studied strains was performed in parallel at UHCZ and DSMZ. The isolates grown on a solid medium underwent protein extraction as previously described and identification using MALDI Biotyper Mycobacteria Library v6.0 with MALDI Biotyper Microflex LT/SH (Bruker Daltonics GmbH, Bremen, Germany) (O’Connor et al., 2016; Alcaide et al., 2018). The Bacterial Test Standard (Bruker Daltonics GmbH, Bremen, Germany) was used for calibration. The MALDI-TOF analysis did not provide reliable identification using the mycobacterial library mentioned above. In addition, the profiles were analyzed in 24 technical replicates using flexAnalysis 3.4 software. According to the literature and the thresholds used by the manufacturer, scores of ≥1.80 and 1.60 to 1.79 represented high confidence and low confidence identification, respectively. A score of <1.60 is considered unreliable (Rodriguez-Temporal et al., 2020).

DNA extraction was performed by resuspending a loop-full of a bacterial colony in 100 μL of distilled water (AccuGENE, Lonza, Belgium), followed by incubation at 95°C for 20 min with shaking at 350 rpm (Thermomixer comfort, Eppendorf). After centrifugation at 14,000 g for 1 min (SL8, Thermo Scientific, Germany), the supernatant was used as a DNA template for PCR.

Genus identification of strains CVI_P3^T^ and CVI_P4 was performed using two genus-specific PCR assays for mycobacteria (Supplementary Figures S8, S9). The first assay amplified the *hsp65* gene (encoding the 65-kDa heat shock antigen) with primers TB1 (5′-GAG ATC GAG CTG GAG GAT CC-3′) and TB2 (5′-AGC TGC AGC CCA AAG GTG TT-3′) with an expected product size of 383 bp (Hance et al., 1989). The second assay amplified the 16S rRNA gene with primers 16S rRNA F (5′-ACG GTG GGT ACT AGG TGT GGG TTT C-3′) and 16S rRNA R (5′-TCT GCG ATT ACT AGC GAC TCC GAC TTC A-3′) with an expected product size of 564 bp (Devulder et al., 2005).

For further identification, the studied strains were tested with GenoType Mycobacterium CM (Hain Lifescience, Germany), a commercial molecular genetic assay for the identification of clinically relevant mycobacterial species from cultured material.

For whole-genome sequencing (WGS), DNA was extracted using the NucleoSpin Microbial Mini DNA Kit (Macherey-Nagel, Germany). DNA libraries were prepared using the NEBNext Ultra DNA Sample Prep Master Mix Kit (NEB). Paired-end (2 × 150 bp) sequencing was performed on the NextSeq 6000 System (Illumina) to a minimum coverage of 170×. Genome assembly was performed using Shovill version 1.0.9^1^ with SPAdes version 3.13.1 (Bankevich et al., 2012) as the assembler. Assembly quality was assessed using Quast version 5.0.2 (Gurevich et al., 2013). The EDGAR 3.0 platform (Dieckmann et al., 2021) was used to construct nucleotide and amino acid alignments of the core genome comprising 1,198 core genes. The nucleotide and the amino acid-based core genome phylogeny were constructed using RAxML version 8.2.12 (Stamatakis, 2014) with the maximum-likelihood method. Average nucleotide identity values based on the MUMmer algorithm (ANIm) were calculated using pyani version 0.2.11 (Pritchard et al., 2016). For 16S rRNA gene phylogeny, the complete 16S rRNA gene sequences of strains CVI_P3^T^ and CVI_P4 were extracted from the annotated draft genomes and aligned using Clustal Omega.

## 3 Results and discussion

Five healthy individuals of the noble pen shell were collected a few weeks before the MMEs in Mljet National Park (Croatia) in 2019, one for each sampling site. Samples of digestive glands, mantles and gills were subjected to bacteriological examination. Growth was detected on the slants seeded with samples of gills and a mantle of a single adult noble pen shell (53 cm long, 18.5 cm wide and 6.6 cm thick) collected at sampling site 1 (Supplementary Figure S1), resulting in the acquisition of strains CVI_P3^T^ and CVI_P4. Acid-fast bacilli indicative of mycobacteria were subcultured and growth, biochemical and phenotypic characterization were performed (Figure 1). The results of growth rate, ability to grow at different temperatures and colony morphology are summarized and compared with closely related organisms in Table 1.

**Table 1:** Phenotypic characteristics of the studied strains CVI_P3^T^ and CVI_P4 and closely related organisms.

Phenotypic characters	CVI_P3^T^/CVI_ P4	Mycobacterium crocinum*	Mycobacterium pallens*	Mycobacterium aromaticivorans*	Mycobacterium iranicum**
Origin	Pen shell gills	Non-contaminated soil	Non-contaminated soil	Contaminated soil	Various human clinical samples
Pigment production	Yes	Yes	Yes	Yes	Yes
Pigment	Yellow-orange	Yellow-orange	Pale-orange	Yellow-orange	Orange
Pigment classification	Scotochromogenic	Scotochromogenic	Scotochromogenic	Scotochromogenic	Scotochromogenic
Growth rate	Rapid	Rapid	Rapid	Rapid	Rapid
Colony morphology	Smooth	Smooth	–	Smooth	Smooth
Cell morphology	Rod-shaped	Long thin rods	Medium rods	Coccoid and long rod shapes	–
Ziehl Nielsen	Positive	Positive	Positive	Positive	Positive
Gram staining	Weakly positive	Weakly positive	Weakly positive	Weakly positive	Positive
Temperature of growth (°C)	25–37°C, optimal at 28°C	28–37°C, optimal at 37°C	28–37°C	28 and 37°C	25–40°C, optimal at 37°C
NaCl tolerance	Yes, in aerobic conditions	Tolerates 2.5% salinity, not 5%	Negative in 2.5 and 5%	Tolerates 2.5–5.0%	Tolerates 2.5–5.0%
Aerobic growth	Aerobic-microaerophilic	Aerobic	Aerobic	Aerobic	Microaerophilic
Tween 80 hydrolysis	Positive	–	–	–	Negative
Nitrate reduction	Positive	Positive	Positive	Negative	Negative
Urease activity	Negative	Negative	Negative	Positive	Positive
Catalase activity	Negative	Positive	Positive	Positive	Positive

* Hennessee et al. (2009).

** Shojaei et al. (2013).

Mycolic acid analysis was performed on the two strains. Fatty acid names and percentages were calculated using the MIS Standard Software (Microbial ID) and are shown in Supplementary Table S2 and Supplementary Figures S4–S6.

The MALDI-TOF analysis did not provide reliable identification using the mycobacterial library mentioned above. The main spectra profiles (MSPs) generated by FlexAnalysis after smoothing and subtraction are shown in Supplementary Figure S7. According to the literature and the thresholds used by the manufacturer, scores of ≥1,80 and 1,60 to 1,79 represented high and low confidence identification, respectively. A score of <1.60 is considered unreliable (Rodriguez-Temporal et al., 2020). However, MALDI-TOF analysis performed at DSMZ found that the most closely related strain was *M. aurum* DSM 6695 with a score value of 1.37. This strain was isolated from the soil polluted with vinyl chloride in the Netherlands (Hartmans and de Bont, 1992). The most closely matched patterns were also those of *M. pallens* DSM 45404 T DSM b L (score 1.430) and *M. crocinum* DSM 45433 T DSM b L (score 1.220) found by the MALDI-TOF analysis in UHCZ. According to the manufacturer, the matching hints of *M. pallens* and *M. crocinum* were similar to each other. Available data on these two species are scarce. Both were first described from Hawaiian soils in 2009 as rapidly growing mycobacteria, which can degrade polycyclic aromatic hydrocarbons, known organic pollutants (Hennessee et al., 2009).

Antimicrobial susceptibility testing (AST) could not be performed because bacterial growth was insufficient to determine minimum inhibitory concentrations despite several repetitions of the assay.

Genus identification of strains CVI_P3^T^ and CVI_P4 was performed using two genus-specific PCR assays for mycobacteria, namely *hsp65* and 16S rRNA (Supplementary Figures S8, S9). The assays determined the genus but were not specific enough to determine the species.

The studied strains were also tested with GenoType Mycobacterium CM, a commercial hybridization assay. The assay classified strain CVI_P3^T^ into the *M. scrofulaceum*/*M. paraffinium*/*M. parascrofulaceum* group (Supplementary Figure S10).

**Figure 2:**
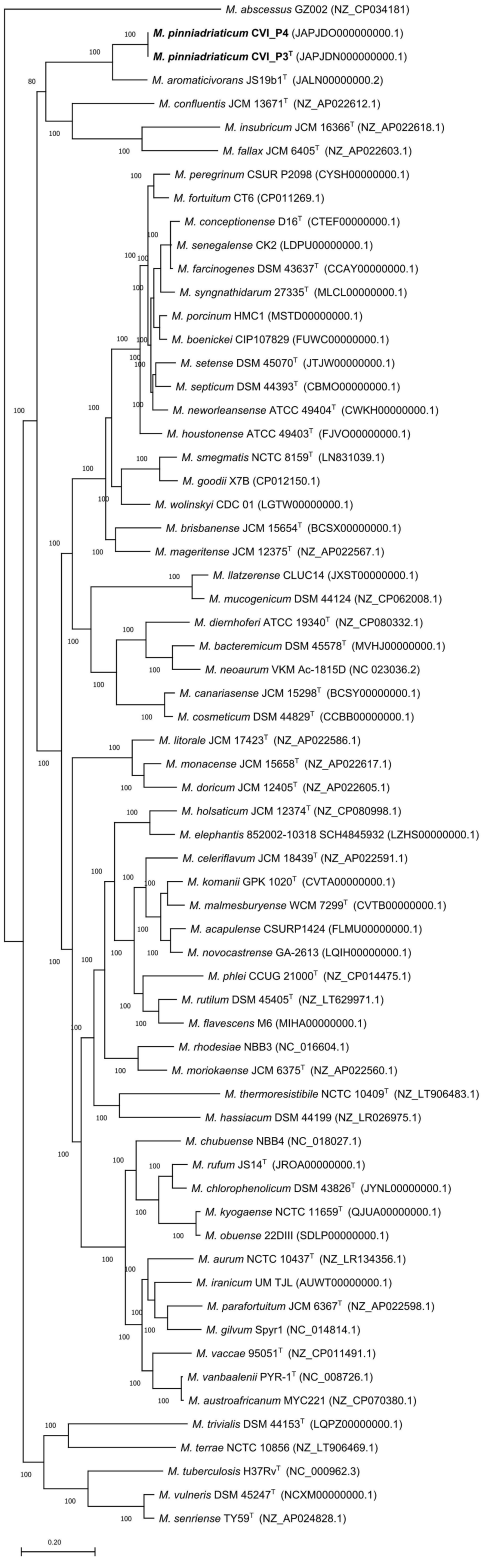
Amino acid-based phylogenetic tree of the core genome showing the phylogenetic position of *Mycobacterium pinniadriaticum* sp. nov. (in bold) within the genus *Mycobacterium*. *M. pinniadriaticum* strains CVI_P3^T^ and CVI_P4 were obtained from the gills and mantle of a noble pen shell, respectively; the shell was collected a few weeks before the mass mortality events in Mljet National Park (Croatia) in 2019. The core genome alignment was constructed using EDGAR 3.0 and comprised 1,198 concatenated core genes and 477,090 amino acid residues per genome. The maximum-likelihood phylogenetic tree was constructed with RAxML version 8.2.12 with the PROTGAMMALGF substitution model. Values on the branches represent bootstrap values. *Mycobacterium abscessus* GZ002 was used as an outgroup and root. Bar, the average number of nucleotide substitutions per site.

Assembly of the whole genome filtered sequences resulted in draft genomes of the two strains, both 6.9 Mb in size and had a G + C content of 66.3% (Supplementary Table S3). The EDGAR platform used to construct nucleotide and amino acid alignments of the core genome found 1,198 core genes. The core genome alignment had 1,457,448 bp and 485,816 amino acid residues per genome. The nucleotide and the amino acid-based core genome phylogeny showed that the studied strains form a separate clade most closely related to *M. aromaticivorans*, previously described in Hennessee et al. (2009) (Figure 2, Supplementary Figure S2).

**Figure 3:**
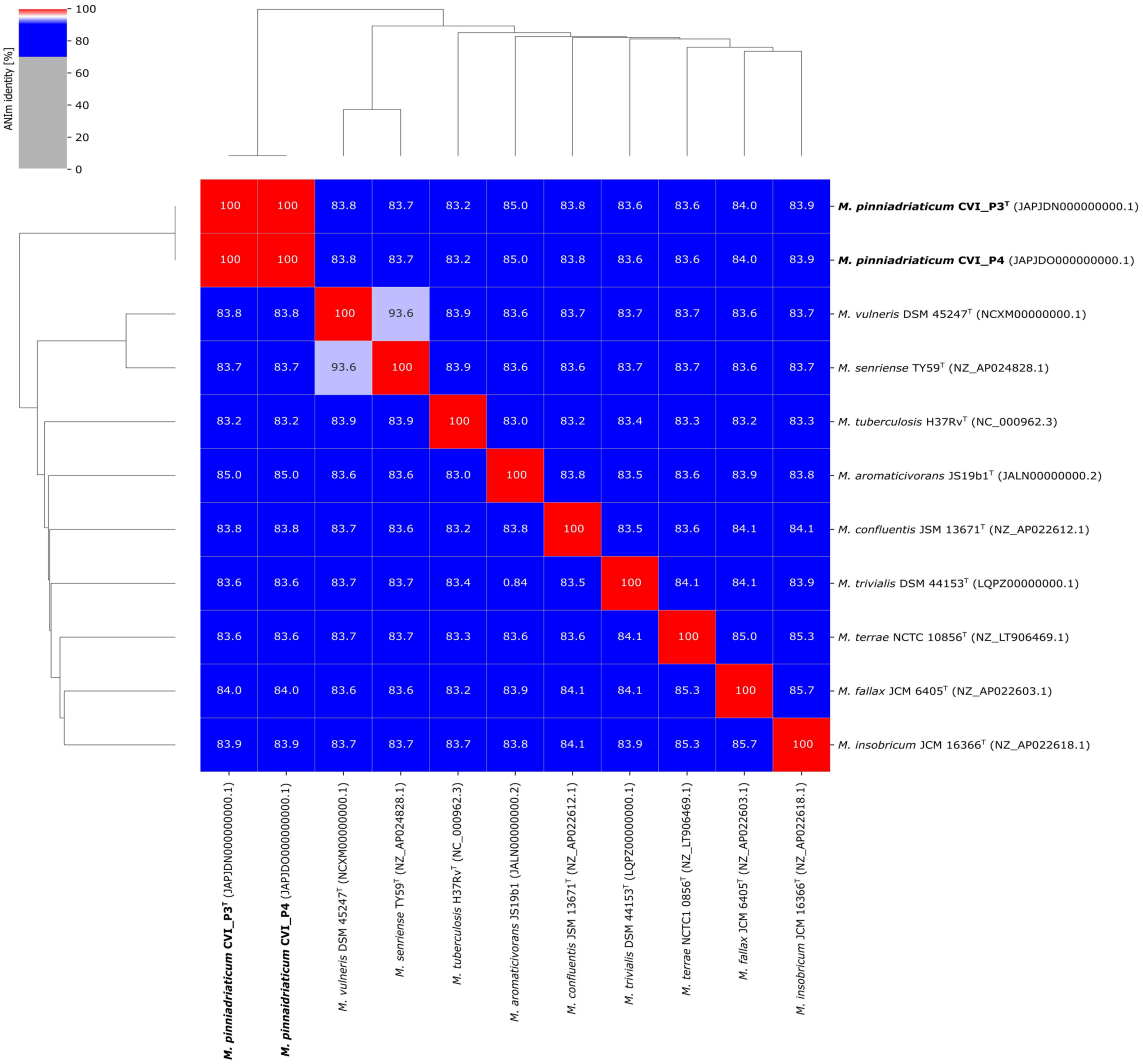
Average nucleotide identity (ANI) values of strains CVI_P3^T^ and CVI_P4 compared with selected closely related mycobacterial species.

A pairwise comparison of the two studied strains revealed a pairwise ANI value of 100%. Pairwise ANI values with the nine selected closely related *Mycobacterium* species are shown in Figure 3. *M. aromaticivorans* JS19b1 had the highest pairwise ANI value of 85.0% with *M. pinniadriaticum*. The calculated ANI values were well below the generally accepted threshold for species delineation of 95–96% ANI (Rosselló-Mora and Amann, 2001; Goris et al., 2007), strongly suggesting that the studied strains are representatives of a new *Mycobacterium* species.

The 16S rRNA gene sequences of both isolates were identical and were phylogenetically most closely related to *M. iranicum* M05T (Shojaei et al., 2013) with a pairwise identity of 98.25% (Supplementary Figure S3). *M. gilvum* SM 35 T (Stanford and Gunthorpe, 1971) was the best match identified by the EzBioCloud 16S identification service (Yoon et al., 2017) with 98.88% identity.

The two *Mycobacterium* strains from two different organs of an adult were isolated a few weeks before the MME of the *P. nobilis* population on the eastern coast of the Adriatic Sea. This is the first description of *Mycobacterium* sp. successfully cultured from *P. nobilis* and described to the species level. Its possible involvement in the recent MMEs in Croatia and several other locations along the Mediterranean Sea remains unclear; therefore, further studies are needed to clarify this issue. This hypothesis is supported by the fact that the seawater temperature at the time of sampling was lower (about 17.2°C) than at the time of the highest mortality rate, which occurred later with the increase in seawater temperature to 26°C (Mihaljević et al., 2021), which is optimal for the growth of bacterial colonies. Lower seawater temperatures may have hindered the growth of mycobacteria and/or the establishment of their pathogenic potential. In addition, the co-occurrence of different pathogens with the greatest influence of *Mycobacterium* and *Haplosporidium* has been previously suggested (Lattos et al., 2020). Other factors such as seawater temperature, salinity, population density, and age of the pen shell population should also be considered (Carella et al., 2020; Lattos et al., 2020).

## 4 Description of *Mycobacterium pinniadriaticum* sp. nov.

*Mycobacterium pinniadriaticum* sp. nov. (pi.ni.ad.ri.ati.kum. L. fem. Adj. pinna, from the name of the bivalve mollusk genus, isolated from a noble pen shell (*Pinna nobilis*); N.L. gen. n. adriaticum, specific epithet of Adriatic Sea).

Generally, rod-shaped, acid-fast, Gram-positive and acid-resistant by Ziehl-Neelsen staining. No spores or filaments were observed by microscopy. This is also the case with its closest relatives which are also rod-shaped with smaller differences considering the size of the rods. The optimal growth temperature is 25–28C in the aerophilic to the microaerophilic atmosphere. Bacteria are unable to grow at 45°C. Its closest relatives grow mostly in an aerobic atmosphere optimally at 37°C. Scotochromogenic, yellowish colonies become visible after 5–7 days on solid media LJ supplemented with pyruvate which is usually the case with its closest relatives. Growth on Stonebrink and LJ supplemented with glycerol was noted in subcultivation. Growth in the presence of 5% NaCl is also observed. The type strain CVI_P3^T^ is negative for urease and catalase activity. Salinity tolerance and urease activity differ between species that are most closely related. However, catalase activity is negative for *Mycobacterium pinniadriaticum* sp. nov. but usually is positive in its closely related species. Additional phenotypic properties are listed in Supplementary Table S1. Prominent fatty acids (>11%) are C18:1ω9c and C16:1ω6c; the mycolic acid pattern is composed of dicarboxy- or dihydroxy-mycolic acids, α-mycolic acids, wax esters and long-chain alcohols. Comparing its fatty acid profiles to those of its closely related species as well as other rapidly growing mycobacteria, the CVI_P3^T^ strain has a unique profile. Its closest relatives *M. aromaticivorans* JS19b1 and *M. insubricum* have the most similar fatty acid profiles both in composition and percentages. Other rapidly growing species differ significantly in fatty acid composition (Table 2).

**Table 2:** Comparison of the whole-cell fatty acid composition of the CVI_P3^T^ and CVI_P4 strains and closely related strains of rapidly growing mycobacteria.

FAME	1	2	3	4	5	6	7	8	9	10	11	12	13
10:0	0.53	0.23	1.87	ND	ND	ND	ND	ND	ND	ND	ND	ND	ND
12:0	0.88	2.19	0.73	ND	ND	ND	ND	ND	ND	ND	ND	ND	ND
14:0	6.00	9.65	6.95	3.4	3.8	5.4	3.7	7.7	5.0	7.8	3.2	4.8	6.0
15:0	0.64	1.10	1.16	<0.4	<0.4	1.1	<0.4	0.5	0.5	0.2	0.3	<0.4	0.3
16:1ω7c + ω6c	13.48	ND	12.20	ND	ND	ND	ND	ND	ND	ND	ND	ND	ND
16:0	23.59	37.92	23.17	19.6	22.4	19.9	21.9	18.2	19.1	23.7	21.7	23.6	34.1
17:0	0.09	0.86	0.51	ND	ND	ND	ND	ND	ND	ND	ND	ND	ND
18:1ω9c	22.79	26.36	29.69	21.4	20.0	23.5	29.4	18.1	23.2	22.2	17.3	32.4	35.5
18:0	1.36	3.24	1.32	1.3	1.1	0.9	2.7	0.6	3.0	2.1	0.9	1.6	1.0
10Me-18:0	8.54	5.02	3.02	16.6	13.6	13.3	10.6	4.7	3.5	8.4	7.5	2.0	3.9
20:0	0.28	1.15	0.84	<0.4	<0.4	<0.4	0.7	0.6	0.5	0.3	<0.4	1.3	<0.4

1. CVI_P3T; 2. *M. insubricum* FI-06250 T; 3. *M. aromaticivorans* JS19b1; 4. *M. murale* MA112/96 T; 5. *M. aurum* NCTC 10437 T; 6. *M. holderi* DSM 44183 T; 7. *M. gadium* NCTC 10942 T; 8. *M. diernhoferi* DSM 43524 T; 9. *M. vaccae* NCTC 10916 T; 10. *M. komossense* ATCC 33013 T; 11. *M. austroafricanum* DSM 44191 T; 12. *M. neoaurum* NCTC 10818 T; 13. *M. abscessus* NCTC 10882.

## Data availability statement

The datasets presented in this study can be found in online repositories. The names of the repository/repositories and accession number(s) can be found in the article/Supplementary material.

## Ethics statement

The animal study was approved by Croatian Ministry of Environmental Protection and Energy. The study was conducted in accordance with the local legislation and institutional requirements.

## Author contributions

SŠ: Conceptualization, Data curation, Funding acquisition, Investigation, Methodology, Project administration, Supervision, Writing – original draft. SD: Investigation, Methodology, Software, Writing – original draft, Writing – review & editing. BP: Data curation, Investigation, Methodology, Software, Visualization, Writing – original draft, Writing – review & editing. IR: Investigation, Methodology, Writing – original draft. SZ: Investigation, Methodology, Writing – original draft. ŽM: Investigation, Methodology, Writing – original draft. ŠN: Investigation, Methodology, Writing – original draft. IZ: Investigation, Methodology, Writing – original draft. GK: Investigation, Methodology, Writing – original draft. BH: Investigation, Methodology, Writing – original draft. IM: Investigation, Methodology, Writing – original draft. MZ-T: Investigation, Methodology, Writing – original draft.

## Footnotes

^1^ https://github.com/tseemann/shovill

## References

[ref1] Alcaide F. Amlerová J. Bou G. Ceyssens P. J. Coll P. Corcoran D.. (2018). European study group on genomics and molecular diagnosis (ESGMD). How to: identify non-tuberculous *Mycobacterium* species using MALDI-TOF mass spectrometry. Clin. Microbiol. Infect. 24, 599–603. doi: 10.1016/j.cmi.2017.11.012, PMID: 29174730

[ref2] Bankevich A. Nurk S. Antipov D. Gurevich A. A. Dvorkin M. Kulikov A. S.. (2012). SPAdes: a new genome assembly algorithm and its applications to single-cell sequencing. J. Comput. Biol. 19, 455–477. doi: 10.1089/cmb.2012.0021, PMID: 22506599 PMC3342519

[ref3] Cabanellas-Reboredo M. Vázquez-Luis M. Mourre B. Álvarez E. Deudero S. Amores A.. (2019). Tracking a mass mortality outbreak of pen shell *Pinna nobilis* populations: A collaborative effort of scientists and citizens. Sci. Rep. 9:13355. doi: 10.1038/s41598-019-49808-4, PMID: 31527825 PMC6746856

[ref4] Carella F. Aceto S. Pollaro F. Miccio A. Iaria C. Carrasco N.. (2019). A mycobacterial disease is associated with the silent mass mortality of the pen shell *Pinna nobilis* along the Tyrrhenian coastline of Italy. Sci. Rep. 9:2725. doi: 10.1038/s41598-018-37217-y, PMID: 30804364 PMC6389904

[ref5] Carella F. Antuofermo E. Farina S. Salati F. Mandas D. Prado P.. (2020). In the wake of the ongoing mass mortality events: co-occurrence of *Mycobacterium*, *Haplosporidium* and other pathogens in *Pinna nobilis* collected in Italy and Spain (Mediterranean Sea). Front. Mar. Sci. 7:48. doi: 10.3389/fmars.2020.00048

[ref6] Čižmek H. Čolić B. Gračan R. Grau A. Catanese G. (2020). An emergency situation for pen shells in the Mediterranean: the Adriatic Sea, one of the last *Pinna nobilis* shelters, is now affected by a mass mortality event. J. Invertebr. Pathol. 173:107388. doi: 10.1016/j.jip.2020.107388, PMID: 32339521

[ref7] Darriba S. (2017). First haplosporidan parasite reported infecting a member of the Superfamily Pinnoidea (*Pinna nobilis*) during a mortality event in Alicante (Spain, Western Mediterranean). J. Invertebr. Pathol. 148, 14–19. doi: 10.1016/j.jip.2017.05.006, PMID: 28511901

[ref8] Devulder G. Perouse de Montclos M. Flandrois J. P. (2005). A multigene approach to phylogenetic analysis using the genus *Mycobacterium* as a model. Int. J. Syst. Evol. Microbiol. 55, 293–302. doi: 10.1099/ijs.0.63222-0 15653890

[ref9] Dieckmann M. A. Beyvers S. Nkouamedjo-Fankep R. C. Hanel P. H. G. Jelonek L. Blom J.. (2021). EDGAR3.0: comparative genomics and phylogenomics on a scalable infrastructure. Nucleic Acids Res. 49, W185–W192. doi: 10.1093/nar/gkab341, PMID: 33988716 PMC8262741

[ref10] Fedrizzi T. Meehan C. J. Grottola A. Giacobazzi E. Fregni Serpini G. Tagliazucchi S.. (2017). Genomic characterization of nontuberculous mycobacteria. Sci. Rep. 7:45258. doi: 10.1038/srep45258, PMID: 28345639 PMC5366915

[ref11] Fogelson S. B. Camus A. C. Lorenz W. Phillips A. Bartlett P. Sanchez S. (2018). *Mycobacterium syngnathidarum* sp. nov., a rapidly growing *Mycobacterium* identified in syngnathid fish. Int. J. Syst. Evol. Microbiol. 68, 3696–3700. doi: 10.1099/ijsem.0.002978, PMID: 30272539

[ref12] García-March J. R. Garcia-Carrascosa A. M. Peña A. L. (2002). *In situ* measurement of *Pinna nobilis* shells for age and growth studies: A new device. Mar. Ecol. 23, 207–217. doi: 10.1046/j.1439-0485.2002.02781.x

[ref13] Goris J. Konstantinidis K. T. Klappenbach J. A. Coenye T. Vandamme P. Tiedje J. M. (2007). DNA–DNA hybridization values and their relationship to whole-genome sequence similarities. Int. J. Syst. Evol. Microbiol. 57, 81–91. doi: 10.1099/ijs.0.64483-0 17220447

[ref14] Gupta R. S. Lo B. Son J. (2018). Phylogenomics and comparative genomic studies robustly support division of the genus *Mycobacterium* into an emended genus *Mycobacterium* and four novel genera. Front. Microbiol. 9:67. doi: 10.3389/fmicb.2018.00067, PMID: 29497402 PMC5819568

[ref15] Gurevich A. Saveliev V. Vyahhi N. Tesler G. (2013). QUAST: quality assessment tool for genome assemblies. Bioinformatics 29, 1072–1075. doi: 10.1093/bioinformatics/btt086, PMID: 23422339 PMC3624806

[ref16] Hance A. J. Grandchamp B. Lévy-Frébault V. Lecossier D. Rauzier J. Bocart D.. (1989). Detection and identification of mycobacteria by amplification of mycobacterial DNA. Mol. Microbiol. 3, 843–849. doi: 10.1111/j.1365-2958.1989.tb00233.x 2507865

[ref17] Hartmans S. de Bont J. A. M. (1992). Aerobic vinyl chloride metabolism in *Mycobacterium aurum* L1. Appl. Environ. Microb. 58, 1220–1226. doi: 10.1128/aem.58.4.1220-1226.1992, PMID: 1599242 PMC195578

[ref18] Hennessee C. T. Seo J.-S. Alvarez A. M. Li Q. X. (2009). Polycyclic aromatic hydrocarbon-degrading species isolated from Hawaiian soils: *Mycobacterium crocinum* sp. nov., *Mycobacterium pallens* sp. nov., *Mycobacterium rutilum* sp. nov., *Mycobacterium rufum* sp. nov. and *Mycobacterium aromaticivorans* sp. nov. Int. J. Syst. Evol. Microbiol. 59, 378–387. doi: 10.1099/ijs.0.65827-0 19196782

[ref19] Kent P. T. Kubica G. P. (1985) Public Health Mycobacteriology. A Guide for the Level III Laboratory. US Department of Health and Human Services, Public Health Service, Centers for Disease Control: Atlanta, GA.

[ref20] Kersting D. K. García-March J. R. (2017). Long-term assessment of recruitment, early stages and population dynamics of the endangered Mediterranean fan mussel *Pinna nobilis* in the Columbretes Islands (NW Mediterranean). Mar. Environ. Res. 130, 282–292. doi: 10.1016/j.marenvres.2017.08.007 28870538

[ref21] Lattos A. Giantsis I. A. Karagiannis D. Michaelidis B. (2020). First detection of the invasive *Haplosporidian* and *Mycobacteria* parasites hosting the endangered bivalve *Pinna nobilis* in Thermaikos Gulf. North Greece. *Mar. Environ. Res.* 155:104889. doi: 10.1016/j.marenvres.2020.104889, PMID: 32072991

[ref9001] Linnaeus C. (1758). Systema Naturae per regna tria naturae, secundum classes, ordines, genera, species, cum characteribus, differentiis, synonymis, locis. Editio decima, reformata [10th revised edition], Laurentius Salvius: Holmiae 824.

[ref22] Meehan C. J. Barco R. A. Loh Y. H. E. Cogneau S. Rigouts L. (2021). Reconstituting the genus *Mycobacterium*. Int. J. Syst. Evol. Microbiol. 71:004922. doi: 10.1099/ijsem.0.004922, PMID: 34554081 PMC8549266

[ref23] Mihaljević Ž. Pavlinec Ž. Zupičić I. G. Oraić D. Popijač A. Pećar O.. (2021). Noble pen shell (*Pinna nobilis*) mortalities along the Eastern Adriatic coast with a study of the spreading velocity. J. Mar. Sci. 9:764. doi: 10.3390/jmse9070764

[ref24] Musser E. Smith C. Halse T. A. Kohlerschmidt D. Rourke A. Fiero A.. (2022). Characterization of *Mycobacterium salfingeri* sp. nov.: A novel nontuberculous mycobacteria isolated from a human wound infection. Front. Microbiol. 13, 1–10. doi: 10.3389/fmicb.2022.992610, PMID: 36299734 PMC9589434

[ref25] Nouioui I. Brunet L. R. Simpson D. Klenk H. P. Goodfellow M. (2018). Description of a novel species of fast-growing *Mycobacterium*: *Mycobacterium kyogaense* sp. nov. a scotochromogenic strain received as *Mycobacterium vaccae*. Int. J. Syst. Evol. Microbiol. 68, 3726–3734. doi: 10.1099/ijsem.0.003039 30300123

[ref26] O’Connor J. A. Lynch-Healy M. Corcoran D. O’Reilly B. O’Mahony J. Lucey B. (2016). Improved matrix-assisted laser desorption ionization-time of flight mass spectrometry (MALDI-TOF MS)-based identification of *Mycobacterium* spp. by use of a novel two-step cell disruption preparatory technique. J. Clin. Microbiol. 54, 495–496. doi: 10.1128/JCM.02998-15, PMID: 26607981 PMC4733187

[ref27] Öndes F. Kaiser M. J. Güçlüsoy H. (2020). Human impacts on the endangered fan mussel, *Pinna nobilis*. Aquatic conservation. Mar. Freshw. Ecosyst. 30, 31–41. doi: 10.1002/aqc.3237

[ref28] Panarese R. Tedesco P. Chimienti G. Latrofa M. S. Quaglio F. Passantino G.. (2019). *Haplosporidium pinnae* associated with mass mortality in endangered *Pinna nobilis* fan mussels. J. Invertebr. Pathol. 164, 32–37. doi: 10.1016/j.jip.2019.04.005 31026464

[ref29] Parte A. C. Sardà Carbasse J. Meier-Kolthoff J. P. Reimer L. C. Göker M. (2020). List of prokaryotic names with standing in nomenclature (LPSN) moves to the DSMZ. Int. J. Syst. Evol. Microbiol. 70, 5607–5612. doi: 10.1099/ijsem.0.004332, PMID: 32701423 PMC7723251

[ref30] Pritchard L. Glover R. H. Humphris S. Elphinstoneb J. G. Toth I. K. (2016). Genomics and taxonomy in diagnostics for food security: soft-rotting enterobacterial plant pathogens. Anal. Methods 8, 12–24. doi: 10.1039/C5AY02550H

[ref31] Rodriguez-Temporal D. Rodríguez-Sánchez B. Alcaide F. (2020). Evaluation of MALDI biotyper interpretation criteria for accurate identification of nontuberculous mycobacteria. J. Clin. Microbiol. 58, e01103–e01120. doi: 10.1128/JCM.01103-20, PMID: 32719033 PMC7512167

[ref32] Rosselló-Mora R. Amann R. (2001). The species concept for prokaryotes. FEMS Microbiol. Rev. 25, 39–67. doi: 10.1111/j.1574-6976.2001.tb00571.x 11152940

[ref33] Šarić T. Župan I. Aceto S. Villari G. Palić D. De Vico G.. (2020). Epidemiology of noble pen shell (*Pinna nobilis* L. 1758) mass mortality events in Adriatic Sea is characterized with rapid spreading and acute disease progression. Pathogens 9:776. doi: 10.3390/pathogens9100776, PMID: 32977433 PMC7598175

[ref34] Scarpa F. Sanna D. Azzena I. Mugetti D. Cerutti F. Hosseiniporgham S.. (2020). Multiple non-species-specific pathogens possibly triggered the mass mortality in *Pinna nobilis*. Life 10:238. doi: 10.3390/life10100238, PMID: 33066230 PMC7650684

[ref35] Shahraki A. H. Trovato A. Droz S. Haidarieh P. Borroni E. Mirsaeidi M.. (2017). *Mycobacterium aquaticum* sp. nov., a rapidly growing species isolated from haemodialysis water. Int. J. Syst. Evol. Microbiol. 67, 3279–3282. doi: 10.1099/ijsem.0.002103, PMID: 28829035

[ref36] Shojaei H. Daley C. Gitti Z. Hashemi A. Heidarieh P. Moore E. R. B.. (2013). *Mycobacterium iranicum* sp. nov., a rapidly growing scotochromogenic species isolated from clinical specimens on three different continents. Int. J. Syst. Evol. Microbiol. 63, 1383–1389. doi: 10.1099/ijs.0.043562-0, PMID: 22843713

[ref37] Stamatakis A. (2014). RAxML version 8: a tool for phylogenetic analysis and post-analysis of large phylogenies. Bioinformatics 30, 1312–1313. doi: 10.1093/bioinformatics/btu033, PMID: 24451623 PMC3998144

[ref38] Stanford J. L. Gunthorpe W. J. (1971). A study of some fast-growing scotochromogenic mycobacteria including species descriptions of *Mycobacterium gilvum* (new species) and *Mycobacterium duvalii* (new species). Br. J. Exp. Pathol. 52, 627–637. PMID: 5002706 PMC2072389

[ref39] Tiscar P. G. Rubino F. Fanelli G. Paoletti B. Della Salda L. (2019). Mass mortality of the fan mussel *Pinna nobilis* in Apulia (Ionian Sea) caused by *Haplosporidium pinnae*. Rapport Commission International pour l’exploration scientifique de la Mer Mediterranée 42:30.

[ref40] Tortoli E. Baruzzo S. Heijdra Y. Klenk H. P. Lauria S. Mariottini A.. (2009). *Mycobacterium insubricum* sp. nov. Int. J. Syst. Evol. Microbiol. 59, 1518–1523. doi: 10.1099/ijs.0.003459-0, PMID: 19502346

[ref41] Vázquez-Luis M. Álvarez E. Barrajón A. García-March J. R. Grau A. Hendriks I.. (2017). SOS *Pinna nobilis*: A mass mortality event in Western Mediterranean Sea. Front. Mar. Sci. 4:220. doi: 10.3389/fmars.2017.00220

[ref42] Vilchèze C. Jacobs W. R. (2007). Isolation and analysis of *Mycobacterium tuberculosis* mycolic acids. Curr. Protoc. Microbiol. Chapter 10:Unit 10A.3. doi: 10.1002/9780471729259.mc10a03s05 18770604

[ref43] Vuorio R. Andersson M. A. Rainey F. A. Kroppenstedt R. M. Kämpfer P. Busse H. J.. (1999). A new rapidly growing mycobacterial species, *Mycobacterium murale* sp. nov., isolated from the indoor walls of a children’s day care centre. Int. J. Syst. Bacteriol. 49, 25–35. doi: 10.1099/00207713-49-1-25 10028244

[ref44] Yoon S. H. Ha S. M. Kwon S. Lim J. Kim Y. Seo H.. (2017). Introducing EzBioCloud: a taxonomically united database of 16S rRNA gene sequences and whole-genome assemblies. Int. J. Syst. Evol. Microbiol. 67, 1613–1617. doi: 10.1099/ijsem.0.001755, PMID: 28005526 PMC5563544

[ref45] Zavodnik D. Hrs-Brenko M. Legac M. (1991). “Synopsis on the fan shell *Pinna nobilis* L. In the eastern Adriatic Sea” in Les Espècies Marines à protéger en Méditerranée. eds. Boudouresque C. F. Avon M. Gravez V. (Marseille: GIS Posidonie publications), 169–178.

[ref46] Zhang D. F. Chen X. Zhang X. M. Zhi X. Y. Yao J. C. Jiang Y. (2013). *Mycobacterium sediminis* sp. nov. and *Mycobacterium arabiense* sp. nov., two rapidly growing members of the genus *Mycobacterium*. Int. J. Syst. Evol. Microbiol. 63, 4081–4086. doi: 10.1099/ijs.0.050567-0, PMID: 23728378

