# Supplementary material for: Description of Mycobacterium pinniadriaticum sp. nov., isolated from a noble pen shell (Pinna nobilis) population in Croatia
Source: Front Microbiol. 2023 Dec 15;14:1289182. doi: 10.3389/fmicb.2023.1289182 (PMC10773828; doi:10.3389/fmicb.2023.1289182)
Supplement: Supplementary file 1 [file Table_1.pdf]

Table S1. Phenotypic characteristics of the studied strains CVI_P3^T and CVI_P4

Bacteriological investigation		Colony morphology and growth rate	Cell morphology	Ziehl Nielsen / Gram staining	Temperature range of growth	NaCl tolerance	Aerobic-Microaerophilic-Anaerobic growth	Tween 80 hydrolysis	Nitrate reduction	Urease activity	Catalase activity
**Strain**	**Origin**										
**CVI_P3T**	Pen shell gills	Smooth colonies, yellow-orange, shiny, visible after 5–7 days on solid media LJ with pyruvate, LJ with glycerol and Stonebrink	Rod-shaped	Positive/ weakly positive	25–37°C, optimal at 28°C	Grows only in aerobic conditions with shaking after 6 days at 28°C on M.1509 media	Aerobic and microaerophilic	Positive only under microaerophilic conditions after 7 days, at 28°C, not under anaerobic conditions	Positive under microaerophilic condition, on 28°C, after 7 days	Negative under aerobic and microaerophilic conditions after 14 days at 28°C	Negative
**CVI_P4**	Pen shell mantle	Smooth colonies, yellow-orange, shiny, visible after 5-7 days on solid media LJ with pyruvate, LJ with glycerol and Stonebrink	Rod-shaped	Positive/ weakly positive	25–37°C, optimal at 28°C	Grows only in aerobic conditions with shaking after 6 days at 28 °C on M.1509 media	Aerobic and microaerophilic	Positive only in microaerophilic condition after 7 days at 28°C, not under anaerobic conditions	Positive under microaerophilic conditions at 28°C after 7 days	Negative under aerobic and microaerophilic conditions after 14 days at 28°C	Negative
