# Supplementary material for: Description of Mycobacterium pinniadriaticum sp. nov., isolated from a noble pen shell (Pinna nobilis) population in Croatia
Source: Front Microbiol. 2023 Dec 15;14:1289182. doi: 10.3389/fmicb.2023.1289182 (PMC10773828; doi:10.3389/fmicb.2023.1289182)
Supplement: Supplementary file 2 [file Table_2.pdf]

Table S2. Mycolic acid composition of strain CVI_P3^T.

Formula	Calculated [M-H]$^-$	Measured [M-H]$^-$	Relative abundance [%]
**Dicarboxy- or dihydroxy-mycolic acids**			
$C_{55}H_{106}O_5$	845.7962	845.7964	12.26
$C_{56}H_{108}O_5$	859.8119	859.8114	2.32
$C_{57}H_{110}O_5$	873.8275	873.8286	20.64
$C_{58}H_{112}O_5$	887.8432	887.8439	18.44
$C_{59}H_{114}O_5$	901.8588	901.8587	13.01
$C_{60}H_{116}O_5$	915.8745	915.8740	10.54
$C_{61}H_{118}O_5$	929.8901	929.8894	3.13
**α-mycolic acids**			
$C_{72}H_{140}O_3$	1052.0724	1052.0742	6.24
$C_{73}H_{142}O_3$	1066.0881	1066.0882	3.09
$C_{74}H_{144}O_3$	1080.1037	1080.1053	5.00
$C_{75}H_{146}O_3$	1094.1194	1094.1191	3.01
$C_{76}H_{148}O_3$	1108.1350	1108.1319	2.31
