# Supplementary material for: Description of Mycobacterium pinniadriaticum sp. nov., isolated from a noble pen shell (Pinna nobilis) population in Croatia
Source: Front Microbiol. 2023 Dec 15;14:1289182. doi: 10.3389/fmicb.2023.1289182 (PMC10773828; doi:10.3389/fmicb.2023.1289182)
Supplement: Supplementary file 3 [file Table_3.pdf]

Table S3. Assembly metrics for the draft genomes of strains CVI_P3^T and CVI_P4.

Strain	CVI_P3^T	CVI_P4
G+C mol%	66.28	66.27
Total assembly size [Mb]	6.87	6.87
N_{50} [bp]	147,621	147,621
No. of contigs (>500 bp)	122	122
