# Supplementary material for: Description of Mycobacterium pinniadriaticum sp. nov., isolated from a noble pen shell (Pinna nobilis) population in Croatia
Source: Front Microbiol. 2023 Dec 15;14:1289182. doi: 10.3389/fmicb.2023.1289182 (PMC10773828; doi:10.3389/fmicb.2023.1289182)
Supplement: Supplementary file 4 [file Data_Sheet_1.docx]

**Supplementary data**

**Evidence of *Mycobacterium pinniadriaticum* sp. nov., a possible co-pathogen of a mass mortality outbreak of the noble pen shell (*Pinna nobilis*) populations in Croatia**

**Authors:**

Silvio Špičić^1^, Sanja Duvnjak*^1^, Bojan Papić*^2^, Irena Reil^1^, Snježana Zrnčić^3^, Željko Mihaljević^4^, Šimun Naletilić^4^, Ivana Giovanna Zupičić^3^, Gordan Kompes^5^, Boris Habrun^5^, Ivana Mareković^6^, Maja Zdelar-Tuk^1^

**Author affiliations:**

^1^Croatian Veterinary Institute, Department of Bacteriology and Parasitology, Laboratory for Bacterial Zoonosis and Molecular Diagnostics of Bacterial Diseases. Savska street 143, Zagreb, Croatia;

^2^University of Ljubljana, Veterinary Faculty, Institute of Microbiology and Parasitology, Gerbičeva 60, Ljubljana, Slovenia;

^3^Croatian Veterinary Institute, Department for Pathological Morphology, Laboratory for Fish Pathology. Savska street 143, Zagreb, Croatia;

^4^Croatian Veterinary Institute, Department for Pathological Morphology, Laboratory for Pathology. Savska street 143, Zagreb, Croatia;

^5^Croatian Veterinary Institute, Department of Bacteriology and Parasitology, Laboratory for General Bacteriology and Mycology. Savska street 143, Zagreb, Croatia;

^6^University Hospital Centre Zagreb, Department of Clinical and Molecular Microbiology, Kišpatićeva 12, Zagreb, Croatia

***Correspondence**: marjanovic@veinst.hr (SD) and bojan.papic@vf.uni-lj.si (BP)

**Fig. S1.** Pen shells in native habitat in Mljet National Park, Small Lake, April 2019. Geographic coordinates: dd:42 mm:47 ss:2.06 N / dd:17 mm: 20 ss: 58.33 E; Latitude: 42.78390 North; Longitude: 17.34956.


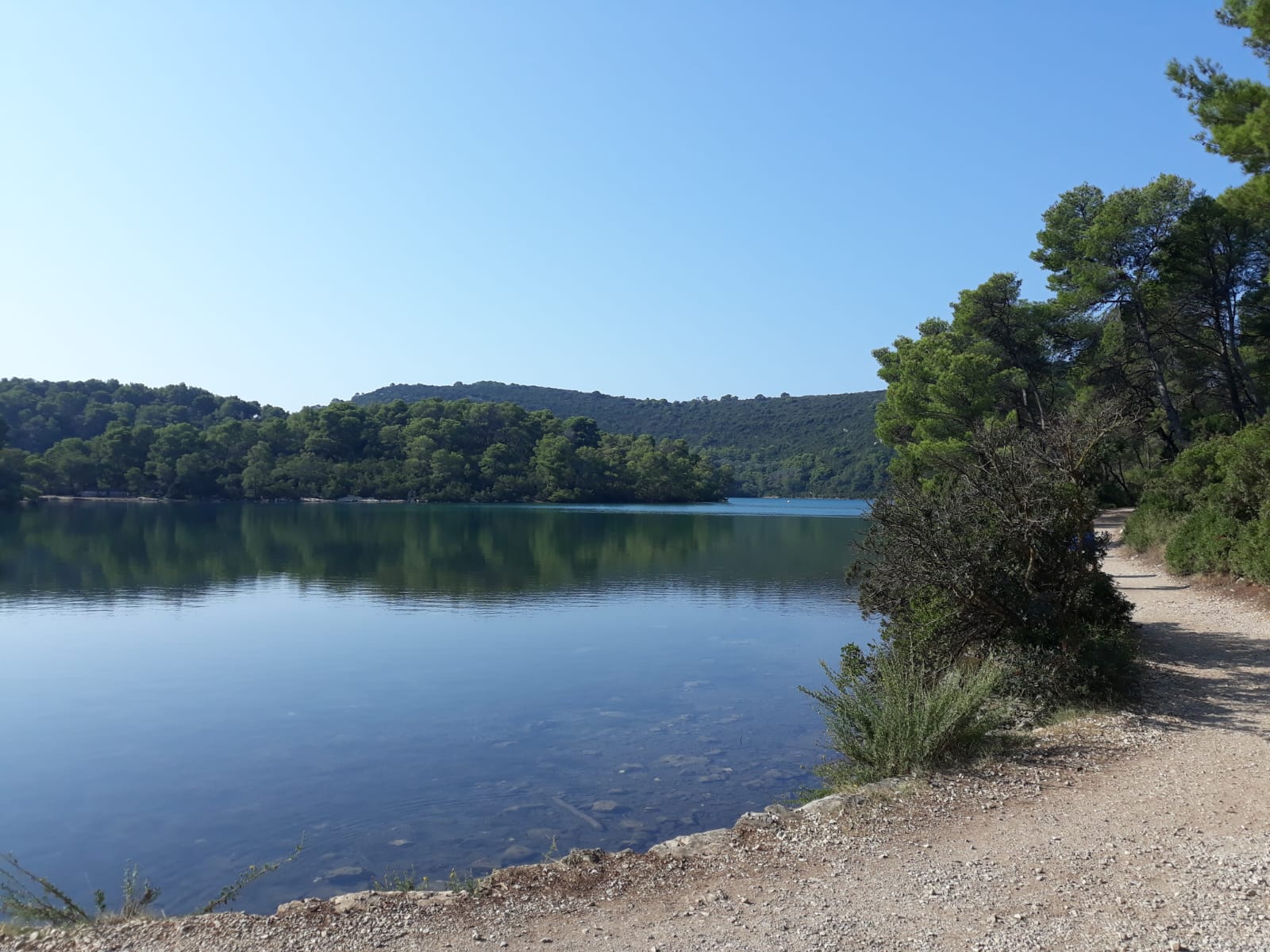


**Fig. S2.** Nucleotide-based phylogenetic tree of the core genome showing the phylogenetic position of Mycobacterium pinniadriaticum sp. nov. (in bold) within the genus Mycobacterium. The core genome alignment was constructed using EDGAR 3.0 and comprised 1198 concatenated core genes and 1,457,448 nucleotide positions per genome. The maximum-likelihood phylogenetic tree was constructed using RAxML version 8.2.12 with the GTRGAMMA substitution model. Values on the branches represent bootstrap values. Mycobacterium abscessus GZ002 was used as an outgroup and root. Bar, the average number of nucleotide substitutions per site.

**Fig. S3.** Maximum-likelihood phylogenetic tree based on 16S rRNA gene sequences. The tree shows the phylogenetic position of Mycobacterium pinniadriaticum sp. nov. (in bold) within the genus Mycobacterium. The alignment of 16S rRNA gene sequences (1571 bp) was performed using Clustal Omega and the phylogenetic tree was generated using RAxML version 8.2.12. Values on the branches represent bootstrap values; only bootstrap values above 70% are shown. Nocardia farcinica DSM 43665T was used as an outgroup and root. Bar, the average number of nucleotide substitutions per site.

**Fig. S4.** Chromatographic run showing the unique fatty acid profile of strain CVI_P3^T^.


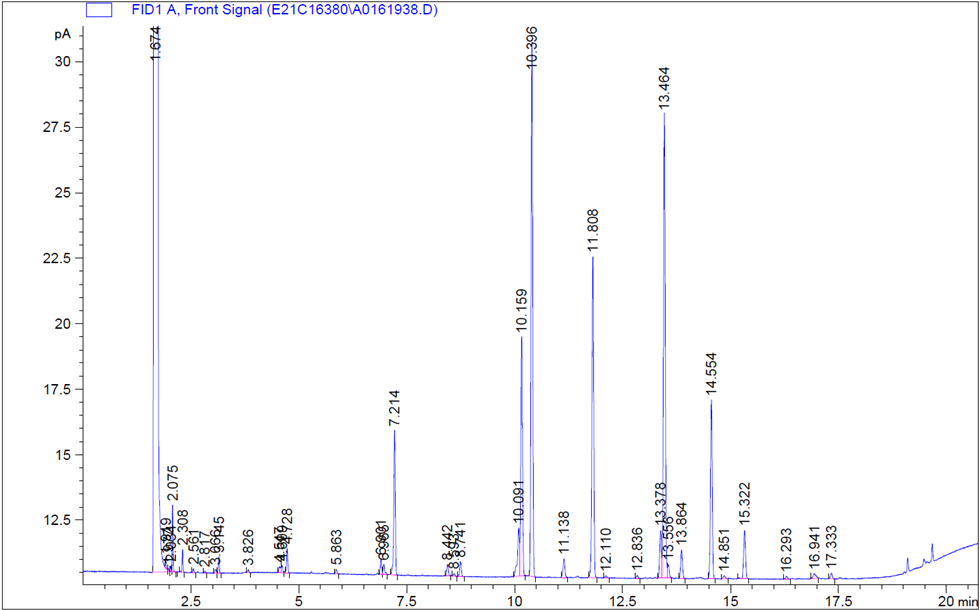


**Fig. S5.** Fatty acid composition of strain CVI_P3^T^


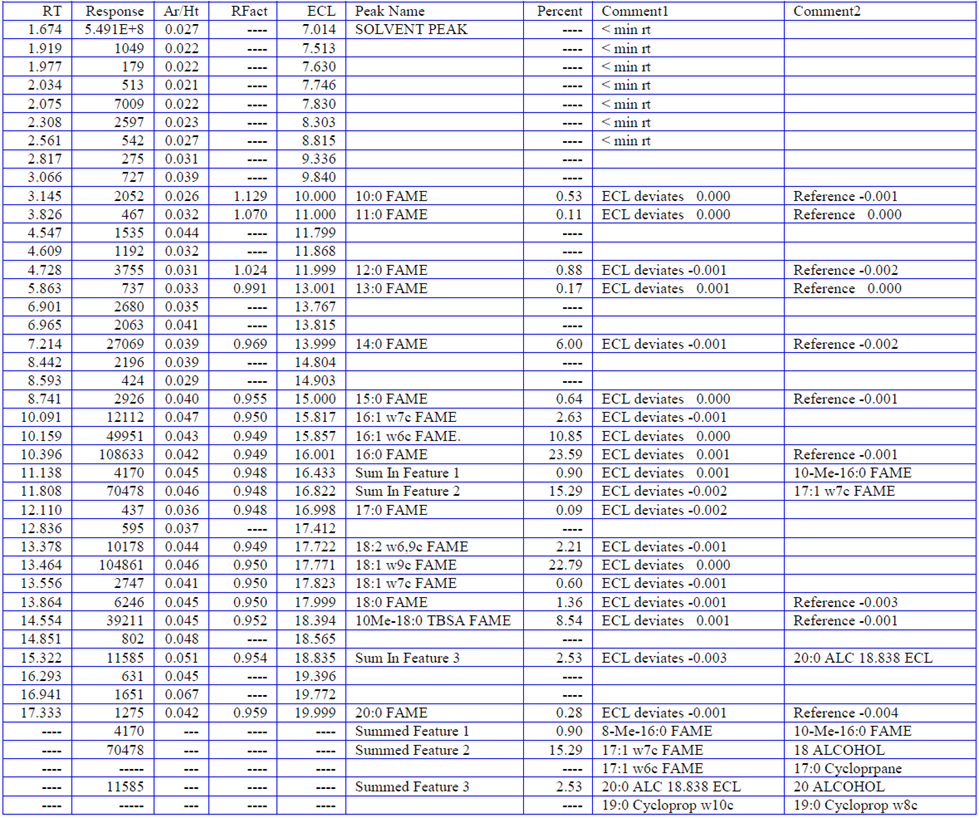


ECL Deviation: 0.001

Reference ECL Shift: 0.002; Number Reference Peaks: 10; Total response: 473662; Total named: 458892; Percent Named: 96.88%; Total Amount: 436974

Matches: *Mycobacterium -scrofulaceum** (MAIS complex): Sim Index 0.082

*Mycobacterium aurum*: Sim Index 0.073

**Fig. S6.**  Fatty acid comparison chart of strain CVI_P3^T^.


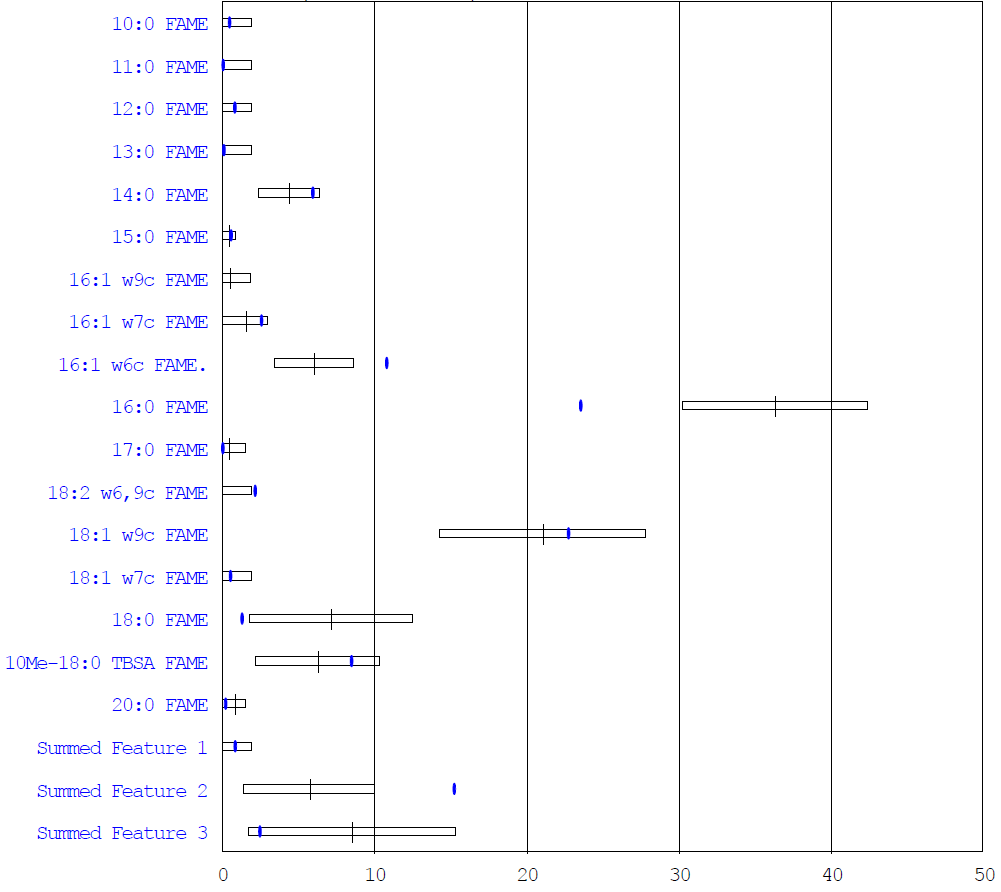


I – mean of the population; **I** – strain CVI_P3^T^; - variance

*Mycobacterium scrofulaceum** (MAIS complex): Sim index 0.082 (Distance: 8.471)

Similarity **(Sim) index** of the Sherlock Microbial Identification System (MIS) is a numerical value, which expresses how closely the fatty acid composition of an unknown organism compares with the mean fatty acid composition of the strains used to create the library entry listed as its match. The perfect mean percentage for all fatty acids in a single species entry (no variance in any of the fatty acids) is indicated by the line at the centre. The Sim index for a strain that falls on this line is 1.000. Values lower than 0.300 suggest that the species is not present in the database, but the software will still indicate the most closely related species.

**Fig. S7.** MALDI-TOF main spectrum (MSP) of strain CVI_P3^T^, which was obtained by processing 24 mass spectra with the flexAnalysis software. m/z represents mass divided by charge number.


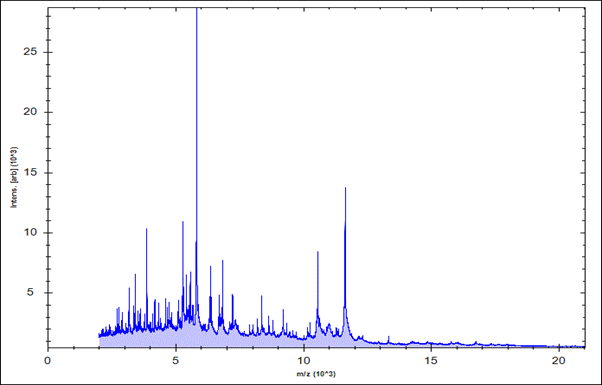


**Fig. S8.** Confirmation of genus *Mycobacterium* based on the amplification of 16S rRNA gene. The amplification products were analysed by QIAxcel capillary electrophoresis (Qiagen, Hilden, Germany) using 50 bp – 1.5 kb QX DNA size marker (Qiaxcel, Hilden, Germany).


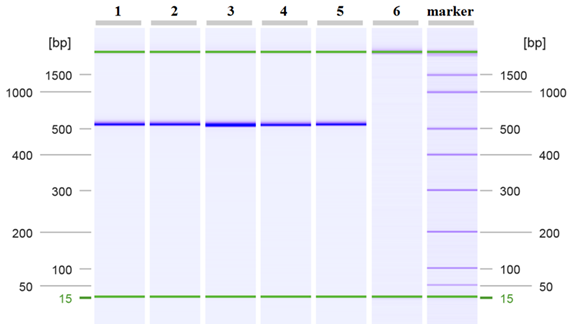


1. strain CVI_P3^T^; 2. strain CVI_P4; 3. positive control – *M. bovis* Bacillus Calmette-Guérin (BCG) strain ATCC 19015; 4. positive control – *Mycobacterium avium* subsp. *avium* serotype 2 strain ATCC 25291; 5. positive control – *M. fortuitum* (archive strain from the collection of the Croatian Veterinary Institute); 6. negative control

**Fig. S9.** Confirmation of genus *Mycobacterium* based on the amplification of the *hsp65* gene. The amplification products were analyzed by QIAxcel capillary electrophoresis (Qiagen, Hilden, Germany) using 50 bp – 1.5 kb QX DNA size marker (Qiaxcel, Hilden, Germany).


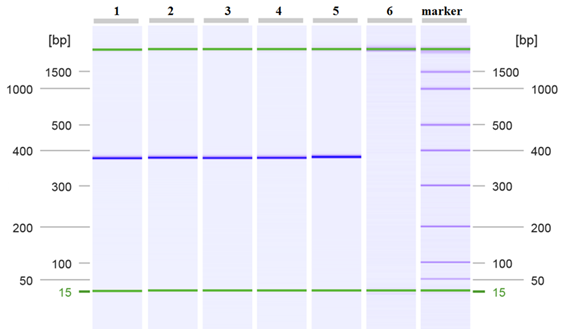


1. strain CVI_P3^T^; 2. strain CVI_P4; 3. positive control – *M. bovis* Bacillus Calmette-Guérin (BCG) strain ATCC 19015; 4. positive control – *Mycobacterium avium* subsp. *avium* serotype 2 strain ATCC 25291; 5. positive control – *M. fortuitum* (archive strain from the collection of the Croatian Veterinary Institute ); 6. negative control

**Figure S10.** Results of the GenoType *Mycobacterium* CM kit for strain CVI_P3^T^


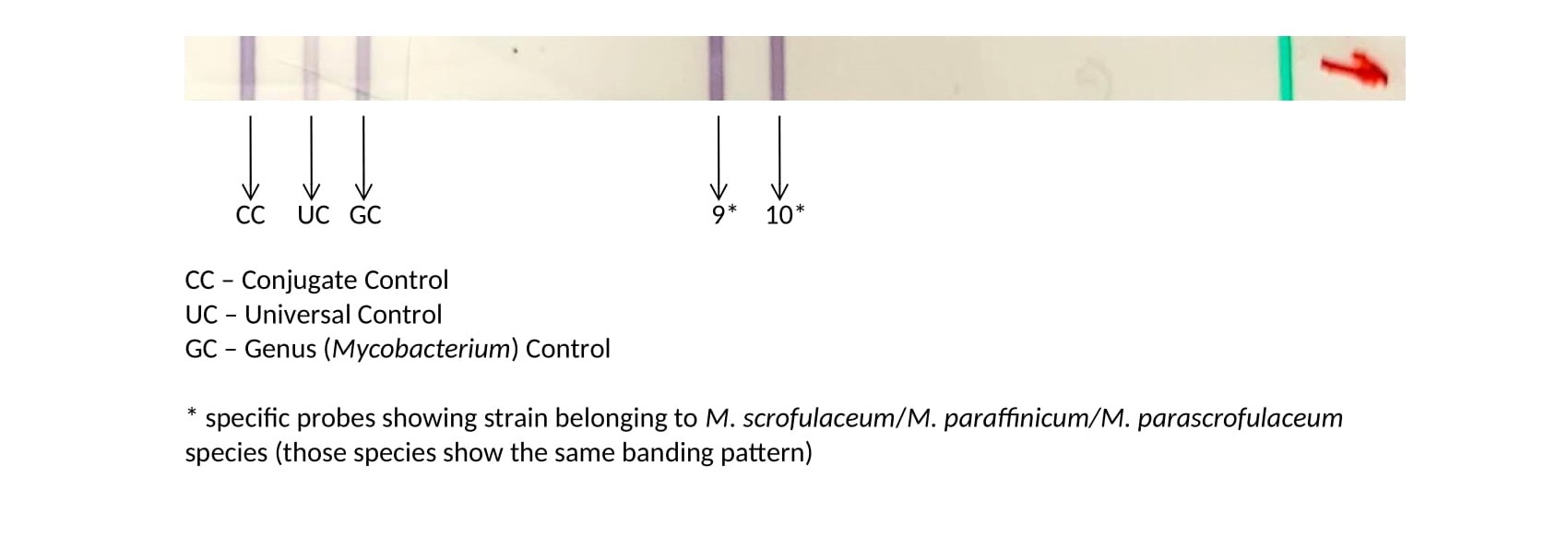


**Table S1.** Phenotypic characteristics of the studied strains CVI_P3^T^ and CVI_P4

| **Bacteriological investigation** | | **Colony morphology and growth rate** | **Cell morphology** | **Ziehl Nielsen / Gram staining** | **Temperature range of growth** | **NaCl tolerance** | **Aerobic- Microaerophilic-Anaerobic growth** | **Tween 80 hydrolysis** | **Nitrate reduction** | **Urease activity** | **Catalase activity** |
| --- | --- | --- | --- | --- | --- | --- | --- | --- | --- | --- | --- |
| **Strain** | **Origin** |  |  |  |  |  |  |  |  |  |  |
| **CVI_P3^T^** | Pen shell gills | Smooth colonies, yellow-orange, shiny, visible after 5–7 days on solid media LJ with pyruvate, LJ with glycerol and Stonebrink | Rod-shaped | Positive/ | 25–37°C, optimal at 28°C | Grows only in aerobic conditions with shaking after 6 days at 28°C on M.1509 media | Aerobic and microaerophilic | Positive only under microaerophilic conditions after 7 days, at 28°C, not under anaerobic conditions | Positive under microaerophilic condition, on 28°C, after 7 days | Negative under aerobic and microaerophilic conditions after 14 days at 28°C | Negative |
|  |  |  |  | weakly positive |  |  |  |  |  |  |  |
| **CVI_P4** | Pen shell mantle | Smooth colonies, yellow-orange, shiny, visible after 5-7 days on solid media LJ with pyruvate, LJ with glycerol and Stonebrink | Rod-shaped | Positive/ | 25–37°C, optimal at 28°C | Grows only in aerobic conditions with shaking after 6 days at 28 °C on M.1509 media | Aerobic and microaerophilic | Positive only in microaerophilic condition after 7 days at 28°C, not under anaerobic conditions | Positive under microaerophilic conditions at 28°C after 7 days | Negative under aerobic and microaerophilic conditions after 14 days at 28°C | Negative |
|  |  |  |  | weakly positive |  |  |  |  |  |  |  |

**Table S2.** Mycolic acid composition of strain CVI_P3^T^.

| **Formula** | **Calculated [M-H]^-^** | **Measured [M-H]^-^** | **Relative abundance [%]** |
| --- | --- | --- | --- |
| **Dicarboxy- or dihydroxy-mycolic acids** | | | |
| C55H106O5 | 845.7962 | 845.7964 | 12.26 |
| C56H108O5 | 859.8119 | 859.8114 | 2.32 |
| C57H110O5 | 873.8275 | 873.8286 | 20.64 |
| C58H112O5 | 887.8432 | 887.8439 | 18.44 |
| C59H114O5 | 901.8588 | 901.8587 | 13.01 |
| C60H116O5 | 915.8745 | 915.8740 | 10.54 |
| C61H118O5 | 929.8901 | 929.8894 | 3.13 |
| **α-mycolic acids** | | | |
| C72H140O3 | 1052.0724 | 1052.0742 | 6.24 |
| C73H142O3 | 1066.0881 | 1066.0882 | 3.09 |
| C74H144O3 | 1080.1037 | 1080.1053 | 5.00 |
| C75H146O3 | 1094.1194 | 1094.1191 | 3.01 |
| C76H148O3 | 1108.1350 | 1108.1319 | 2.31 |

**Table S3.** Assembly metrics for the draft genomes of strains CVI_P3^T^ and CVI_P4.

| **Strain** | **CVI_P3^T^** | **CVI_P4** |
| --- | --- | --- |
| G+C mol% | 66.28 | 66.27 |
| Total assembly size [Mb] | 6.87 | 6.87 |
| *N*_50_ [bp] | 147,621 | 147,621 |
| No. of contigs (>500 bp) | 122 | 122 |
