# Supplementary figures and images for: Description of Mycobacterium pinniadriaticum sp. nov., isolated from a noble pen shell (Pinna nobilis) population in Croatia
Source: Front Microbiol. 2023 Dec 15;14:1289182. doi: 10.3389/fmicb.2023.1289182 (PMC10773828; doi:10.3389/fmicb.2023.1289182)

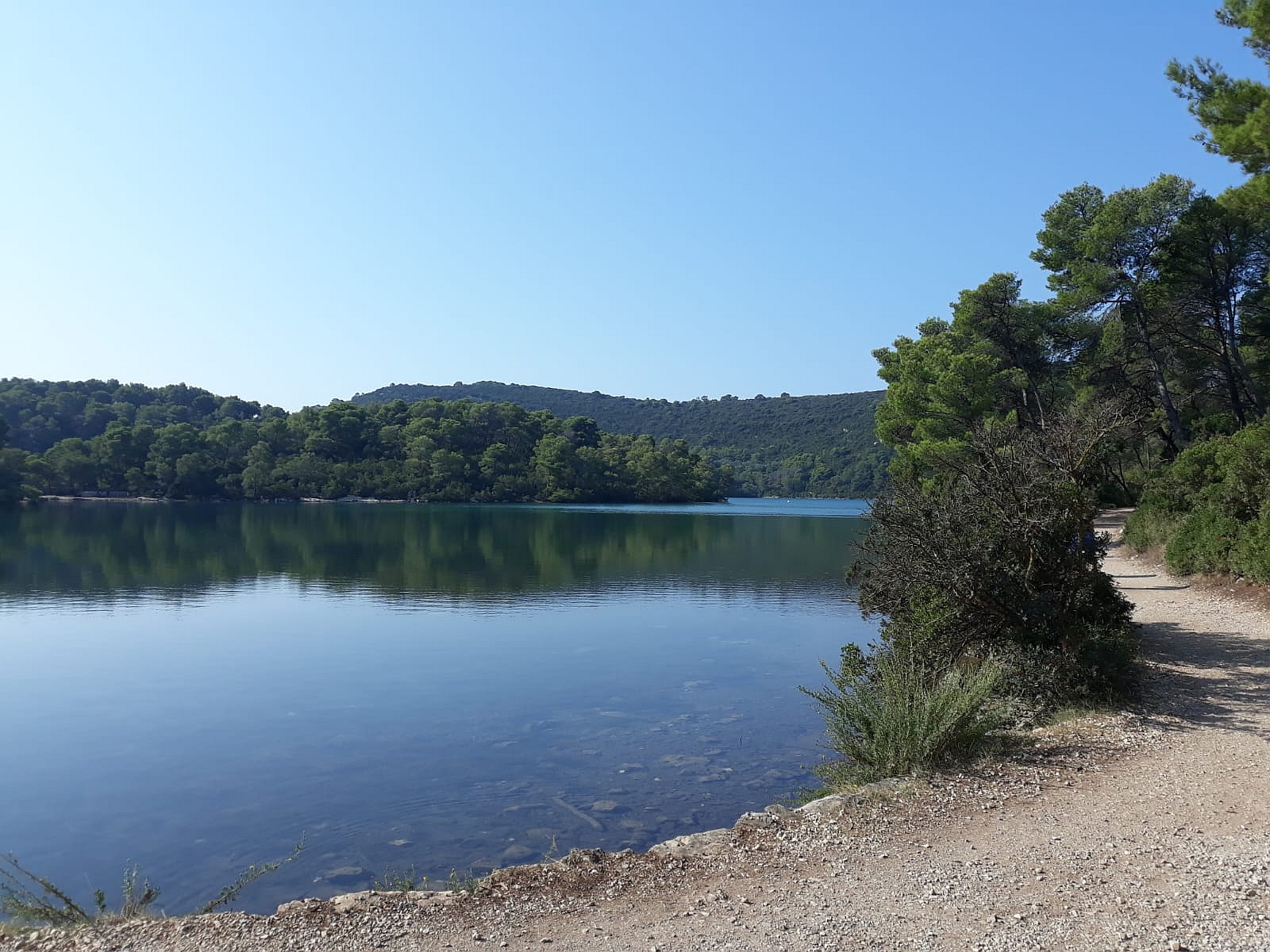

Supplement: Supplementary file 5 [file Image_1.JPEG]

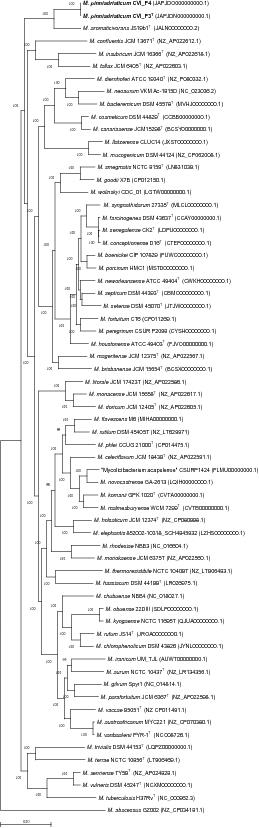

Supplement: Supplementary file 6 [file Image_2.JPEG]

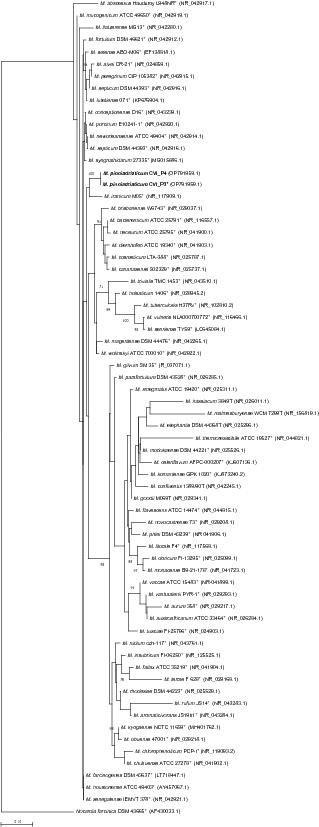

Supplement: Supplementary file 7 [file Image_3.JPEG]

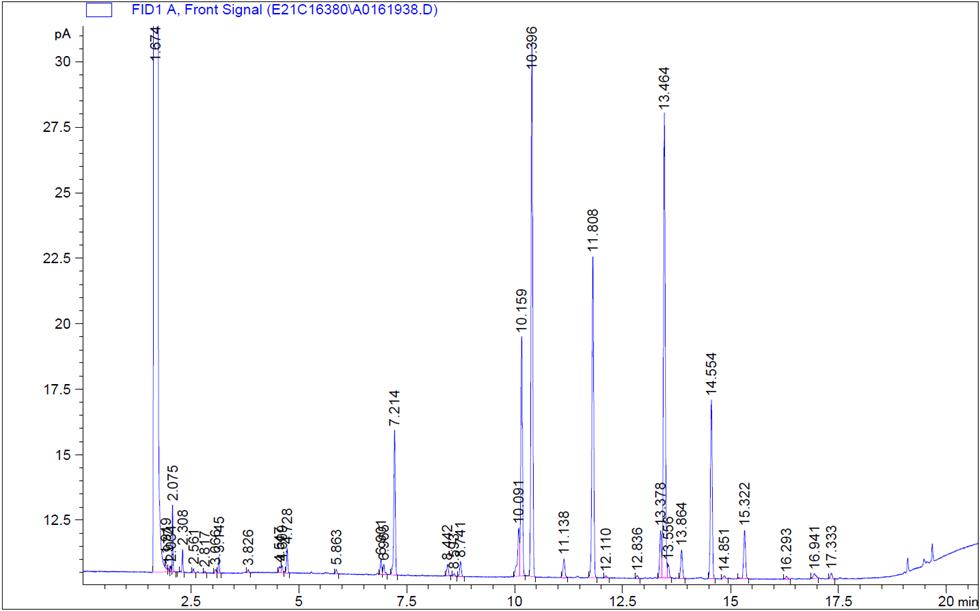

Supplement: Supplementary file 8 [file Image_4.JPEG]

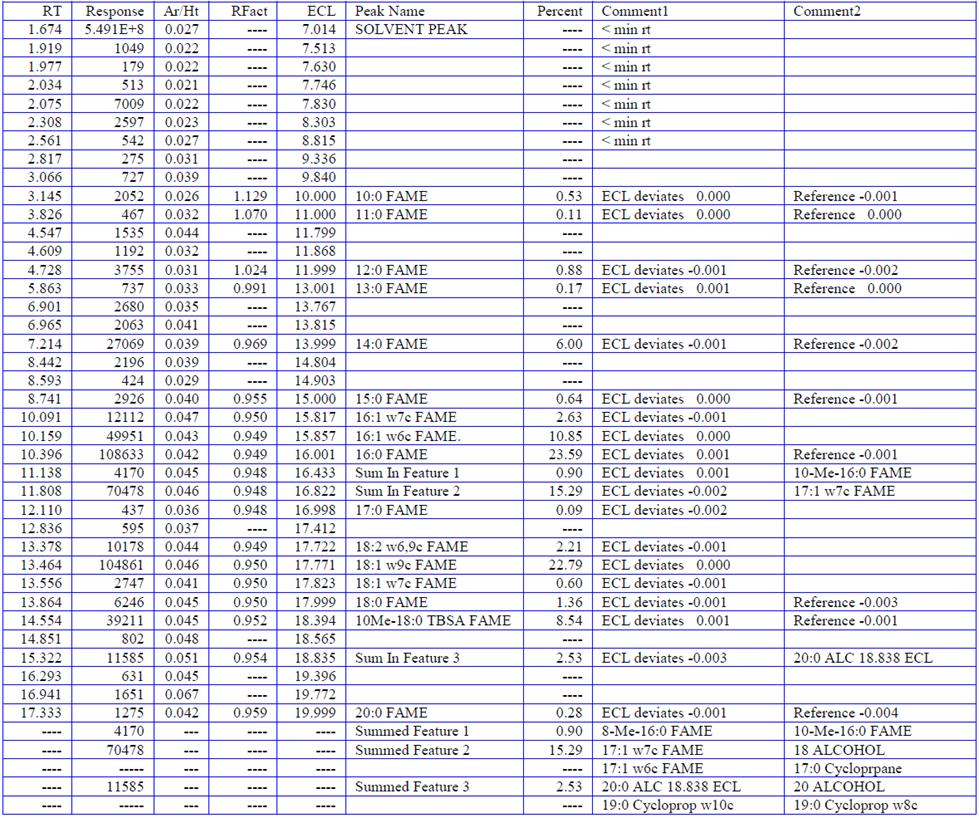

Supplement: Supplementary file 9 [file Image_5.JPEG]

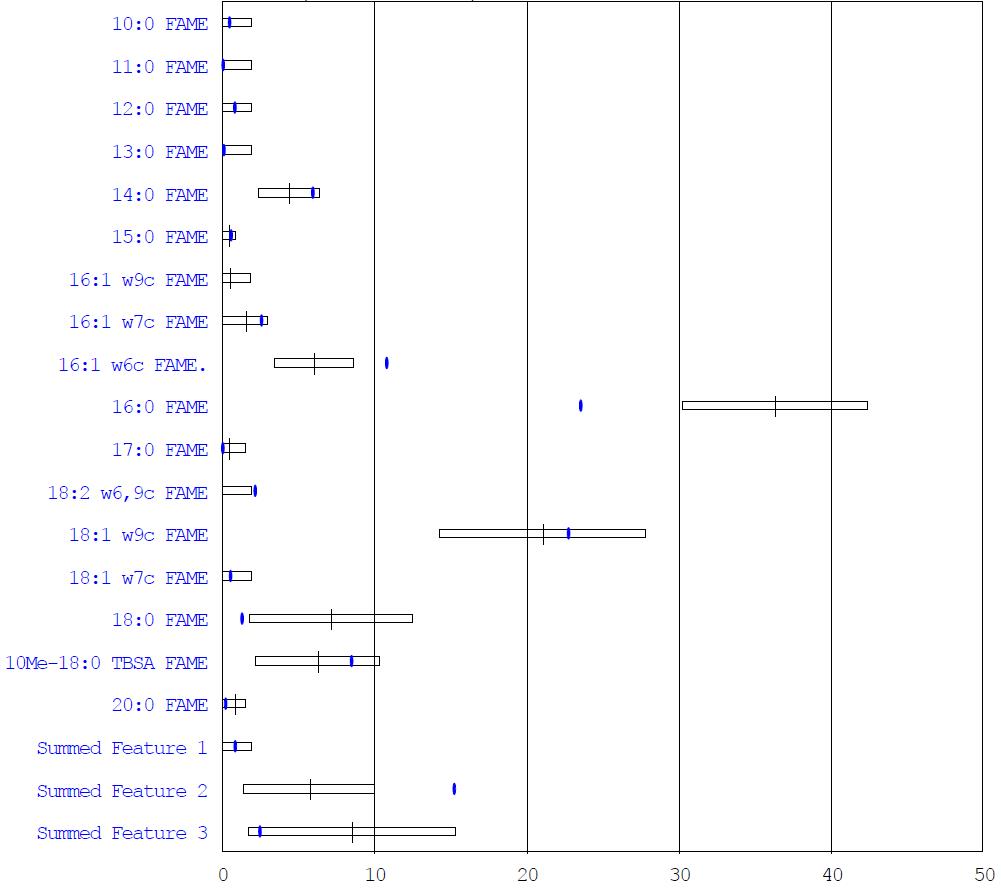

Supplement: Supplementary file 10 [file Image_6.JPEG]

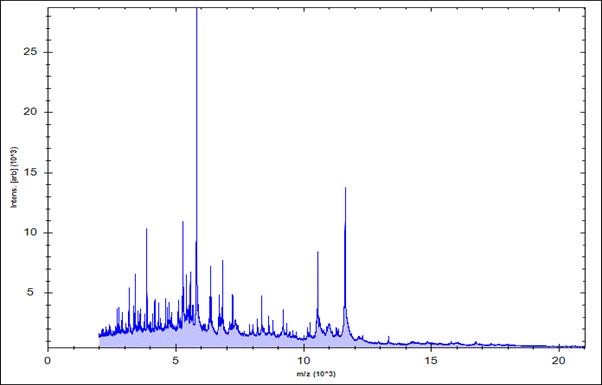

Supplement: Supplementary file 11 [file Image_7.JPEG]

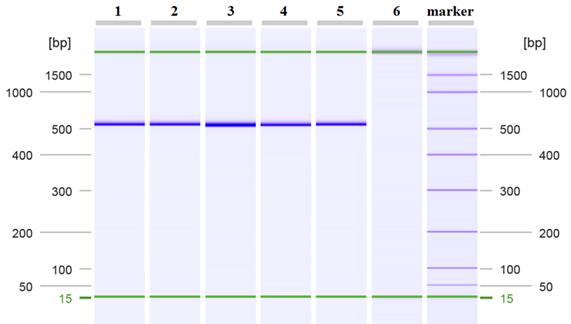

Supplement: Supplementary file 12 [file Image_8.JPEG]

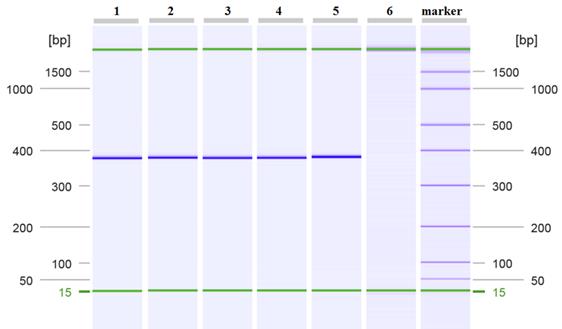

Supplement: Supplementary file 13 [file Image_9.JPEG]

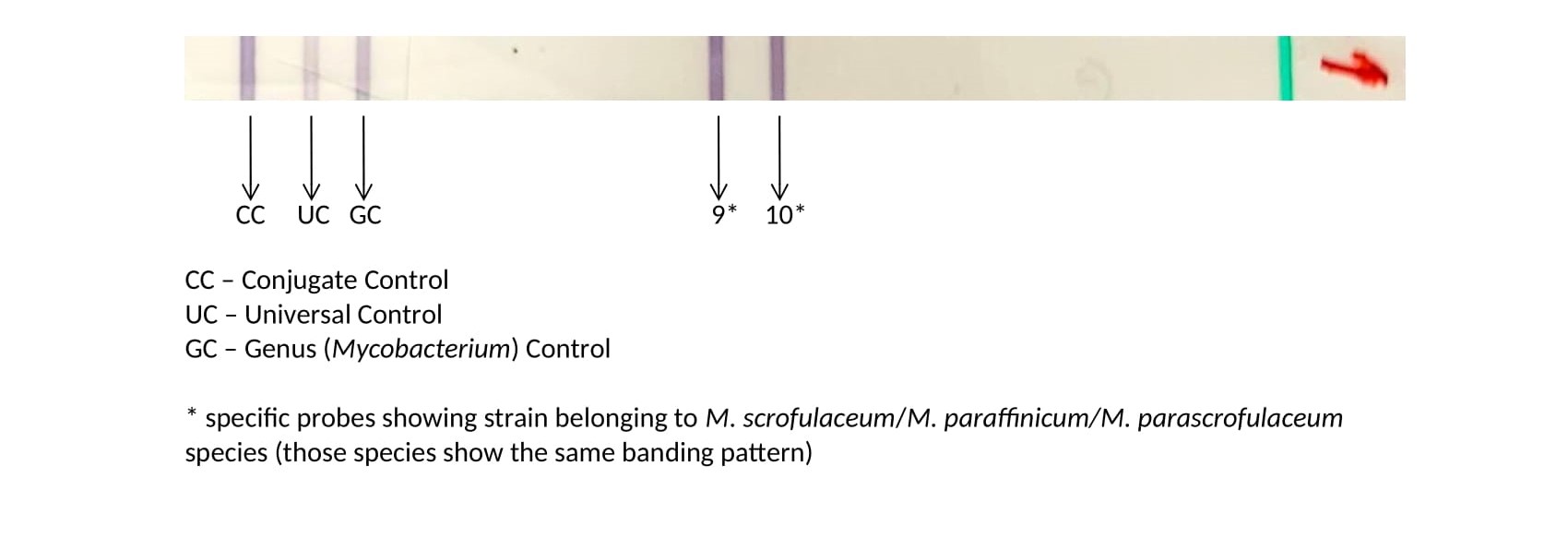

Supplement: Supplementary file 14 [file Image_10.JPEG]
